# Improved *in vivo* optical coherence tomography imaging of animal peripheral nerves using a prism nerve holder

**DOI:** 10.1117/1.JBO.28.2.026002

**Published:** 2023-02-11

**Authors:** Ilyas Saytashev, Yong-Chul Yoon, Benjamin J. Vakoc, Srikanth Vasudevan, Daniel X. Hammer

**Affiliations:** a U. S. Food and Drug Administration, Center for Devices and Radiological Health, Office of Science and Engineering Laboratories, Silver Spring, Maryland, United States; b Massachusetts General Hospital, Wellman Center for Photomedicine, Boston, Massachusetts, United States

**Keywords:** polarization sensitive-optical coherence tomography, peripheral nerve, prism nerve holder, *in vivo* imaging, neurosurgery, birefringence

## Abstract

**Significance:**

Modern optical volumetric imaging modalities, such as optical coherence tomography (OCT), provide enormous information about the structure, function, and physiology of living tissue. Although optical imaging achieves lateral resolution on the order of the wavelength of light used, and OCT achieves axial resolution on a similar micron scale, tissue optical properties, particularly high scattering and absorption, limit light penetration to only a few millimeters. In addition, *in vivo* imaging modalities are susceptible to significant motion artifacts due to cardiac and respiratory function. These effects limit access to artifact-free optical measurements during peripheral neurosurgery to only a portion of the exposed nerve without further modification to the procedure.

**Aim:**

We aim to improve *in vivo* OCT imaging during peripheral neurosurgery in small and large animals by increasing the amount of visualized nerve volume as well as suppressing motion of the imaged area.

**Approach:**

We designed a nerve holder with embedded mirror prisms for peripheral nerve volumetric imaging as well as a specific beam steering strategy to acquire prism and direct view volumes in one session with minimal motion artifacts.

**Results:**

The axially imaged volumes from mirror prisms increased the OCT signal intensity by >22  dB over a 1.25-mm imaging depth in tissue-mimicking phantoms. We then demonstrated the new imaging capabilities in visualizing peripheral nerves from direct and side views in living rats and minipigs using a polarization-sensitive OCT system. Prism views have shown nerve fascicles and vasculature from the bottom half of the imaged nerve which was not visible in direct view.

**Conclusions:**

We demonstrated improved OCT imaging during neurosurgery in small and large animals by combining the use of a prism nerve holder with a specifically designed beam scanning protocol. Our strategy can be applied to existing OCT imaging systems with minimal hardware modification, increasing the nerve tissue volume visualized. Enhanced imaging depth techniques may lead to a greater adoption of structural and functional optical biomarkers in preclinical and clinical medicine.

## 1 Introduction

Linear imaging modalities, such as confocal microscopy and optical coherence tomography (OCT), provide depth-resolved capability for *in vivo* imaging at tissue and cellular scales. For many applications, such as retinal imaging in ophthalmology, light penetration depth is adequate to glean sufficient information for clinical decision-making. However, as OCT expands to new applications in opaque tissues, light scattering and absorption can more adversely limit its utility. One such area is peripheral nerve imaging, where optical imaging modalities have been applied to collect important information about peripheral nervous system (PNS) conditions and the interaction of devices to relieve those conditions (e.g., neuromodulation devices).^1^^–^^4^ For example, vagus nerve stimulation (VNS) is a technique used to treat diseases and conditions, such as epilepsy and depression.^5^ However, the success rate and effectiveness of neurostimulation in humans are highly variable.^6^ Several factors contribute to the overall VNS safe-and-effective use profile, including electrical stimulation parameters (amplitude, frequency, pulse width, duration of stimulation, etc.) and optimal positioning of electrodes for targeted stimulation of nerve fascicles, which may reduce the potential for off-target effects.^7^^,^^8^ OCT angiography (OCTA) and polarization sensitive OCT (PS-OCT) imaging-based biomarkers have been demonstrated for assessment of peripheral nerve damage and overstimulation by providing *in vivo* volumetric structural and functional information.^1^^,^^9^ Our team has introduced custom nerve holders to provide a motion-free platform for *in vivo* peripheral nerve examination.^1^^,^^2^ However, except in the rare case of imaging nerves that are less than a few hundred micrometers in diameter, these OCT-based PNS imaging studies have been unable to demonstrate full-depth imaging of peripheral nerves.

**Fig. 1:**
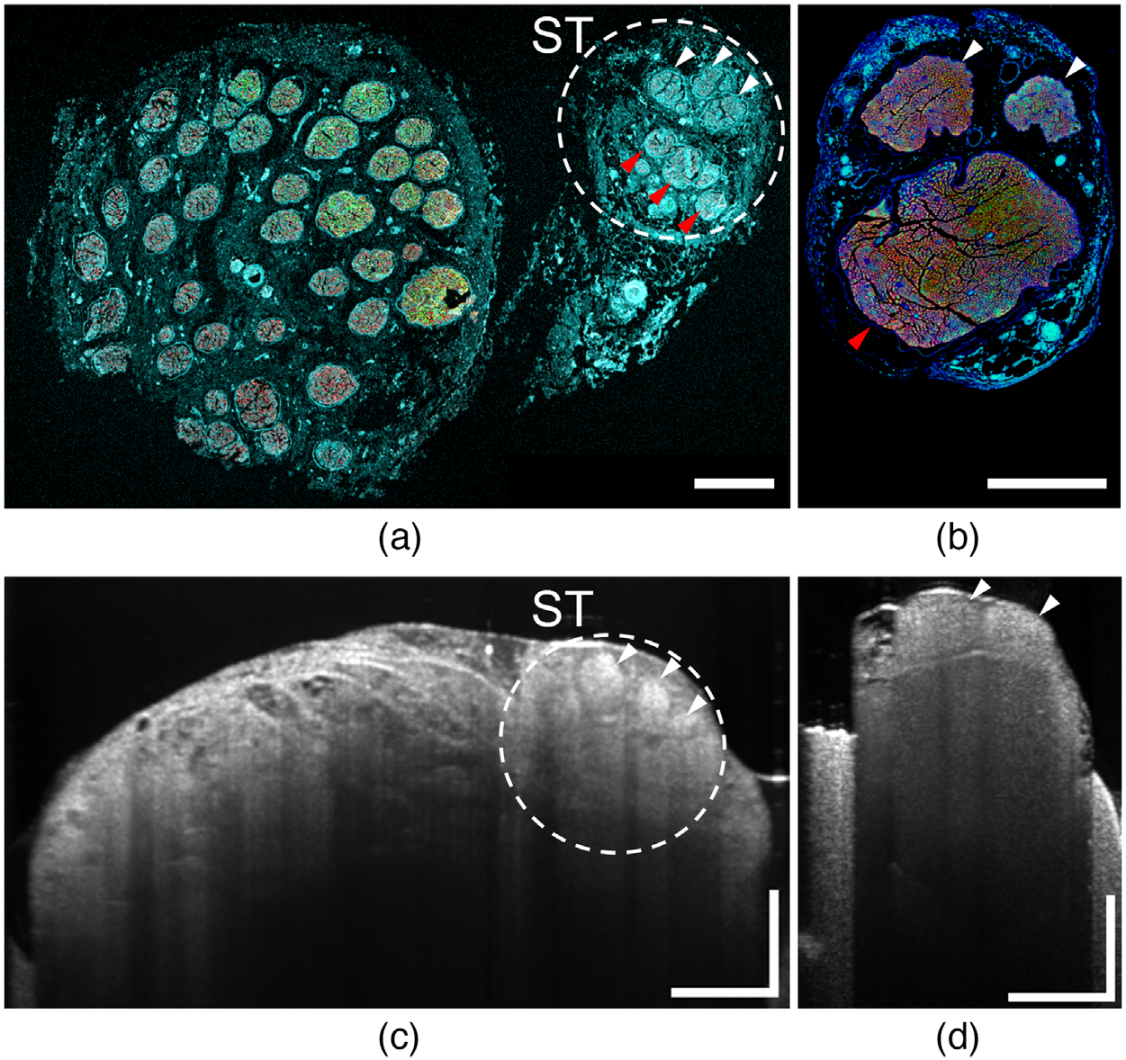
(a), (c) Minipig vagus nerve; (b), (d) rat sciatic nerve histological sections and structural OCT cross sections (B-scans). Examples of nerve fascicles are marked with white and red arrows. Fascicles in minipig sympathetic trunk and rat sciatic nerve are clearly seen on the IHC image [(a), (b): white and red arrows], whereas (c) and (d) OCT B-scans do not provide clear information about fascicles deep within the nerve [(a), (b): red arrows]. B-scan of minipig vagus nerve (c) shows significant proportion of adipose and connective tissue, which hinders the fascicular visualization underneath. (a), (b) IHC images; pseudocolors represent: blue, cell nuclei and connective tissue; green, tubulin; red, myelin. Scale bar: 500  μm.

Typical OCT images of small animal peripheral nerve (rat sciatic nerve, 1 to 1.5 mm in diameter) obtained with a 1310-nm swept-source system are unable to resolve features in the bottom half of the nerve (Fig. 1). For large animal peripheral nerve imaging, the visualization of fascicular and vascular structures becomes even more challenging due to epineurium and adipose connective tissue layers, which can be a few hundred micrometers in thickness. In addition, when an animal’s head position creates a tilt in vagus nerve (VN) propagation—the cranial and caudal ends of a nerve within an OCT volume may be mismatched by as much as 1 mm in depth. Together with OCT image roll-off, this leads to blurred and uneven structural OCT images within a volume or unresolved fine vasculature in OCTA.

A number of techniques have been proposed to increase OCT imaging quality by extending the optical depth-of-field, including beam shaping to produce Bessel,^10^ Bessel-like,^11^ and needle beams.^12^ In general, these techniques use additional hardware (phase plates, multimodal fibers segments, etc.) in the beam path to produce a nondiffracting beam with an extended focus. Although these methods provide improved lateral resolution across the depth of a target, the penetration depth does not improve due to the underlying limits of light intensity of the imaging beam^13^ and tissue scattering and absorption. Our approach to mitigate depth penetration losses is to multiplex light delivery via custom designed nerve holders with side-imaging prisms.

The idea of using a mirror prism for illumination and detection in the axial plane was first demonstrated in confocal microscopy^14^ and then used in submicrometer scale superresolution microscopy.^15^^,^^16^ We have evolved this approach for mesoscopic imaging capable of visualizing several centimeters of peripheral nerve within one image field of view (FOV) capturing directly imaged and prism-illuminated views within one PS-OCT acquisition volume.

Our custom nerve holder design was motivated by several factors, including application to both small and large animal studies, strategies for their use in a surgical environment, consideration of *in vivo* PS-OCT image acquisition techniques, and potential clinical use for ancillary real-time optical imaging during PNS device implantation in humans. First, we imaged tissue-mimicking phantoms in the nerve holders to verify and optimize performance. OCT subvolumes from side views appearing in-focus ensured the correct choice of size and material of the mirror prisms in small and large animal nerve holders. Then we performed acute PS-OCT imaging of rat sciatic and minipig vagus nerves to characterize and validate *in vivo* performance. In this paper, we demonstrate the suppression of motion during *in vivo* imaging as well as the acquisition of complementary volumetric information through side prism imaging. The platform enhances the information collected during optical imaging of peripheral nerves for a variety of emerging neuromodulation applications.

## 2 Materials and Methods

### 2.1 PS-OCT System for Peripheral Nerve Imaging

For testing the prism nerve holder, we used a swept-source (1310-nm central wavelength and 110-nm bandwidth) PS-OCT system that is more fully described elsewhere.^9^^,^^17^ The system was designed for *in vivo* imaging of sciatic and vagus peripheral nerves in small and large animals and configured for field portability for easy transport between surgical suites or between the laboratory and surgical suite. The optical head is mounted on a S21 Zeiss surgical microscope arm and the OCT processing engine, system computer, and other instrumentation were placed on a mobile cart (Thorlabs, Newton, New Jersey) with optical breadboard (Newport Corporation, Irvine, California). The optical head is tethered to the main OCT engine with two sets of electric cables to control galvanometer scanners and the sample path fiber optic cable.

The optical head and OCT engine are configured so that multiple low-NA objectives (LSM02, LSM03, LSM54, Thorlabs, Newton, New Jersey) can be swapped easily. Most often, the system was equipped with the LSM54 objective which provides a 64-mm working distance, especially to image the nerve in the deep surgical pocket of the minipig neck. The 3-mm beam diameter after the collimator provides near-diffraction limited ∼25  μm spot size measured with a phantom (APL-OP01, Arden Inc., Clearwater, Florida) at the focus of LSM54 objective. The numerical dispersion correction vectors for each objective were calculated following the methodology proposed by Singh et al.^18^ from interferometric signals of a mirror in air.

PS-OCT images are acquired as follows. The system images at 50,000 A-lines per second. The polarization of the A-lines is modulated between two orthogonal states such that adjacent A-lines are always orthogonal. Each A-line generates four interferograms, two of which describe the signal in a first polarization state and the remaining two in a second orthogonal polarization state. The two interferograms within each polarization state are fed to a balanced detector. This creates two voltage signals (one from each polarization state) that are digitized by a two-channel DAQ board (ATS9350, Alazar Technologies Inc., Pointe-Claire, Québec, Canada) at 200 MHz synchronized to an external clock generator, sampling each interferogram with 3072 points. During acquisition, custom software writes sampled interferogram data into memory and displays live previews of the structural and angiographic cross-sectional OCT images. The system uses a custom scanning protocol: one volume consists of 1184 B-scans; each B-scan is reconstructed from 1184 lines divided into 4 backstitched segments of 296 lines.^19^^,^^20^ Each of the four segments is acquired 5 times allowing for intensity averaging and calculation of complex differential variance angiography^21^ with time interval of 12 ms due to bidirectional scanning of galvanometer mirrors. The acquisition time is 122 ms per B-scan, or under 150 s per volume. The FOV varies by changing the amplitude of the scanning pattern from 5  mm×5  mm (small animal) to 15  mm×25  mm (large animal nerve).

After image acquisition, each binary file, which contained the sampled interferograms, is processed by graphics processing unit (GPU)-accelerated (Tesla V100, Nvidia corporation, Santa Clara, California) Python software^22^ which outputs processed PS-OCT data into several image stacks representing the volumetric modalities: structural intensity, angiography, optic axis orientation angle, retardation, degree of polarization uniformity, and the birefringence weighted optic axis (BwOA) representation.^17^ Each A-line is initially discretized into 2048 points providing 5.7  μm per depth layer. For visualization, volumes are cropped and rescaled to maintain homogeneous voxel size.

### 2.2 Prism Nerve Holder Design

Peripheral nerve holders were used to stabilize the nerve from respiratory, cardiac, and general animal motion during imaging, which can corrupt OCT images, especially OCTA images, where even pixel-scale motion of a few microns will add speckle or phase noise to the images. In our previous studies, we demonstrated significant improvement in OCTA imaging when using a nerve holder mounted to the animal stage.^1^ Although this arrangement worked for small animals in an optical table-mounted configuration, installing the optical head on a surgical arm for large animal imaging introduced an additional issue with relative motion between the nerve holder and the optical head. This issue was solved by magnetically mounting the nerve holder directly to the optical head at a fixed distance (equal to the objective working distance) via rods attached to a kinematic stage (KC2-T, Thorlabs, Newton, New Jersey) placed around the objective which in turn provides fine focus and tilt adjustment for the nerve target. In this way, common-mode animal motion or vibrations coupled into the setup perturbs the optical head and nerve holder identically, without changing the distance between the optical head and nerve. This configuration significantly reduced motion artifacts in the OCT images.

**Fig. 2:**
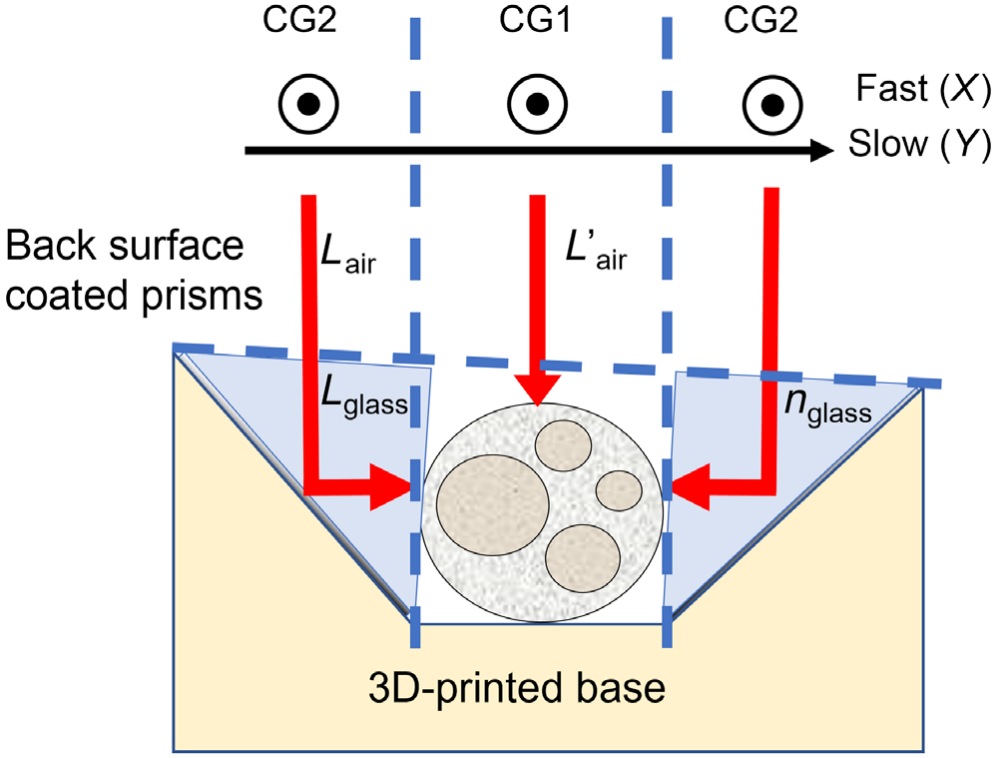
A schematic of the prism nerve holder principle of imaging. The fast-scanning mirror acquisition is aligned along the nerve, whereas the slow-scanning mirror moves perpendicular to the nerve through the first prism, nerve, and the second prism. Respective reference mirror positions are denoted CG2 and CG1 when the beam scans the nerve sides through the prisms or the nerve top directly. Back surface-coated prisms with high-refractive index glass extend the focus and provide beam steering in the axial plane of the nerve.

Figure 1 illustrates light penetration in our peripheral nerve application, specifically the inability of OCT to resolve fascicles and other structures, such as blood vessels in the lower half of the nerve. Our approach to mitigate the effect is simple—scan the side(s) of the nerve with the OCT imaging beam and subdivide the cross-sectional OCT frame to visualize the nerve from one or two sides as well as from the top (Fig. 2). The side scanning can be achieved with a reflecting optic; however, there are several additional issues that need to be considered in the design.

First, the additional optical distance traveled for the side reflected beams means that the distance to the tissue will not match the objective focal length and the tissue will be out of focus, even for a long depth-of-focus objective. This would require inefficient acquisition of two volumes, one focused on the top of the tissue and a second focused on the side(s) after refocus (i.e., axial step). We solved this issue using back-surface reflecting prisms so that the OCT beam passes through the prism glass, extending the focal distance of the side reflected beams to match that of the top directed beam. In this case (Fig. 2), the required focal shift is $\Delta f\approx L_{\text{air}}+L_{\text{glass}}-L_{\mathrm{air}}^{\prime }$. Following the paraxial approximation, the focal shift due to light traveling in glass can also be expressed as $\Delta f\approx L_{\text{glass}}\cdot (1-n_{\text{air}}/n_{\text{glass}})$. Combining these two equations, we get $L_{\text{glass}}\cdot (1-n_{\text{air}}/n_{\text{glass}})\approx L_{\text{air}}+L_{\text{glass}}-L_{\mathrm{air}}^{\prime }$ or, $-L_{\text{glass}}/n_{\text{glass}}\approx L_{\text{air}}-L_{\mathrm{air}}^{\prime }$ considering $n_{\text{air}}\approx 1$. This leads to requirements for the mirror prism glass and size: $n_{\text{glass}}\approx L_{\text{glass}}/(L_{\mathrm{air}}^{\prime }-L_{\mathrm{air}})$.

Several back-surface mirrored prisms with different refractive index glasses were tested to determine the best focus match without significant perturbation in the PS-OCT signal. Back-surface Al-coated mirrored prisms made of N-SF11 glass with AR-coated leg surfaces (Edmund Optics, Barrington, New Jersey) were chosen.

Second, the nerve holder yields a difference in apparent axial image position for the side regions corresponding to beams reflected off the prisms compared to the center region corresponding to the beam directly back-reflected from the nerve due to extended optical path length. Because all OCT systems experience some roll off in signal intensity with axial position, if the difference in the apparent axial positions is large, there can be a significant difference in the OCT signal intensity for the prism-reflected and direct back-reflected regions of the image. However, because the coherence range gate (CG) on our PS-OCT system is electronically controlled, it is possible to compensate this in the software and move the CG during a volume acquisition. Inherent timing jitter means the CG can only be adjusted slowly, and this adjustment is best done by switching the fast and slow axis so that the scanning is done along the prism regions first (CG2), then along the center region (CG1), and then adjusted back to CG2 for the other prism.

Third, the design minimized specular reflections coupled back into the OCT signal by adding a small angular tilt to 3D-printed based on which the back-surface coated prisms were mounted and using antireflective coating on the prisms. As mentioned above, the optical head was also fit with a kinematic stage for moving the nerve axially into the focal plane and a tip-tilt to account for any angle in the nerve within the holder. The design considerations provide optimal conditions for peripheral nerve imaging in small and large animals.

### 2.3 Prism Nerve Holder Fabrication and Assembly

**Fig. 3:**
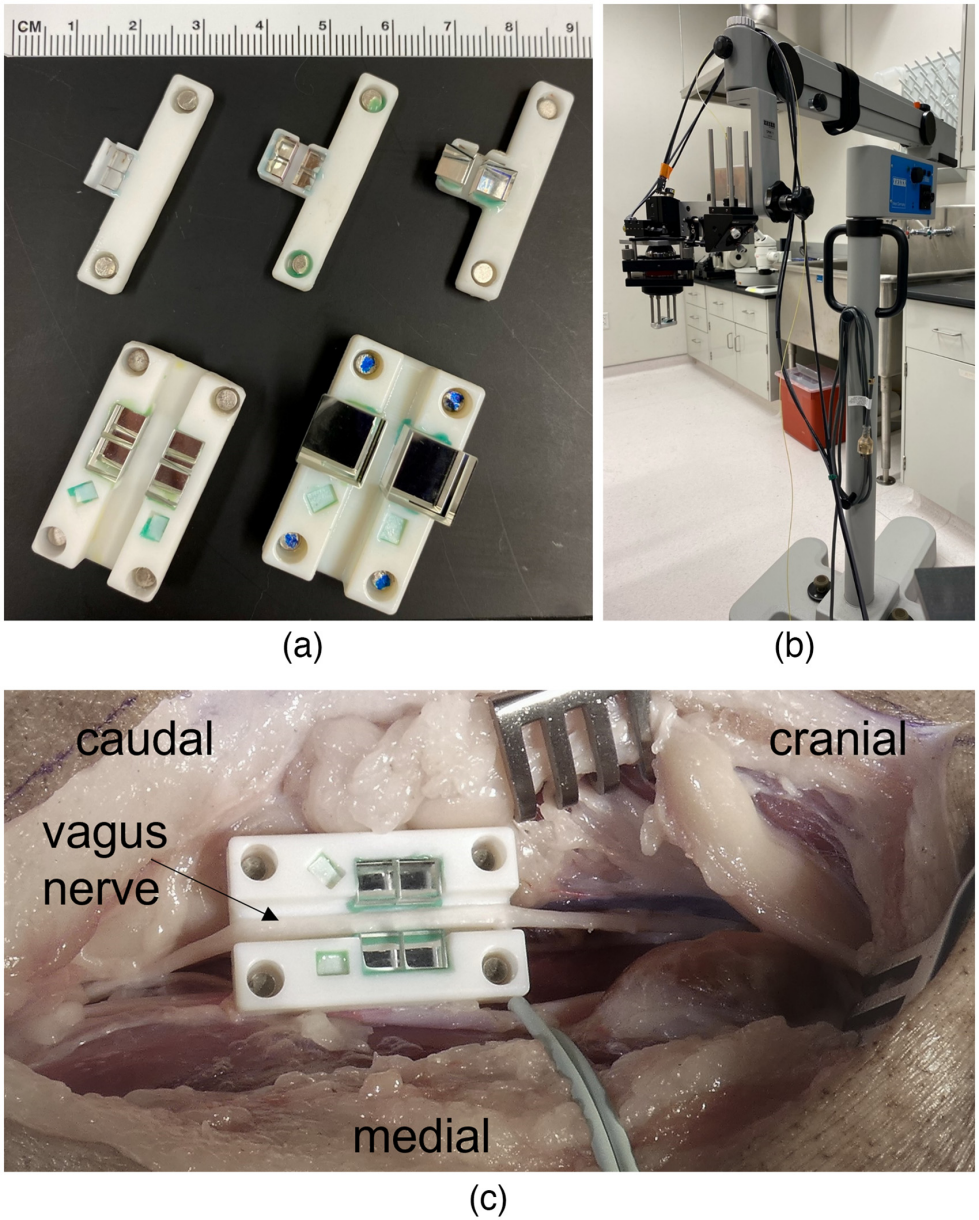
(a) Prism nerve holders for small and large animal peripheral nerve and tissue mimicking phantom imaging. From left to right and top to bottom: magnetic single-sided prism, two magnetic double-sided prism small animal nerve holders with 3 and 5 mm prisms; magnetic double-sided prism large animal nerve holders with 5 and 10 mm prisms. Larger prisms compensate for extended optical path length differences when imaging rigid samples. PS calibration phantoms^23^ were embedded in large animal magnetic prism nerve holders for optical axis orientation calibration. (b) PS-OCT microscope assembly with large animal prism nerve holder attached to the optical head magnetically. The kinematic stage around the microscope objective allows for fine focus and tilt adjustments. (c) Photograph of a minipig neck surgical pocket with vagus nerve placed in a large animal nerve holder with 5 mm prisms during cadaver experiment.

CAD modeling (Solidworks 2018, Dassault, France) and additive manufacturing technology allowed us to rapidly design and fabricate several compact nerve holder bases for imaging tissue-mimicking phantoms, and small and large animal peripheral nerves *in vivo* within the size constraint set by the surgical pocket [Fig. 3(a)]. The design of a nerve holder channel was driven by peripheral nerve anatomy (U-shape, length, and width of the channel, flanged edges). Prism pads were designed to accommodate two 3-mm (small animal) or 5-mm (large animal) prisms along the side of the nerve with the prism edge set below the nerve channel to ensure optical path accessibility to the lowest part of the nerve. Standard magnetic pad cavity placement in the small and large nerve holders allows interchangeable operation with the same optical head hardware.

Either Objet260 Connex3 (Stratasys Ltd., Eden Prairie, Minnesota) or Form 3 (FormLabs Inc., Somerville, Massachusetts) printers were used to fabricate nerve holders. Both printers provided sub-$50\times 50\times 50\;{\mu m}^{3}$ voxel resolution to ensure precise nerve channel dimensions and fit of the prisms. For final assembly, the prisms were carefully placed on the nerve holder base and secured by small drop of soft silicone adhesive in the nonimaged corners of the prisms.

Cylindrical Nd magnets (diameter 1.2 mm and height 1 mm) were glued into the magnet cavities with the correct polarity according to the respective magnetic rod of the optical head. The final assembled nerve holder magnetically attached to optical head on the Zeiss S21 surgical microscope arm is shown in Fig. 3(b). A photograph of a minipig neck surgical pocket during cadaver experiment [Fig. 3(c)] demonstrates an example of placement of a vagus nerve into the large animal nerve holder with 5 mm prisms.

The different versions of the nerve holder allow maximum flexibility for various potential applications. The magnetic prism nerve holders for small animals [Fig. 3(a), top row] were made in a single-sided prism configuration, as well as a 5-mm dual side prism version. Single-side prism versions allow for easier placement of the nerve holder in a limited surgical pocket. Unlike freshly excised or *in vivo* nerve tissue, the mechanical rigidity of a fixed nerve or a phantom preserves the circular shape in cross section during imaging. This leads to an increased difference in the focal length between the prism image and direct image. Therefore, we manufactured versions of the small and large animal nerve holders with larger prisms (5 and 10 mm) to account for increased focal lengths during phantom bench-top imaging.

### 2.4 Prism Nerve Holder Validation Experiments

The prism nerve holder allows the OCT beam to probe nerve regions not normally accessible with direct top-down beam delivery. To aid nerve holder optimization and demonstrate initial operation and performance with a controlled target, we imaged tissue-mimicking phantoms with 1.2- and 3.5-mm cross-sectional diameters. Both phantoms were constructed from polydimethylsiloxane (PDMS) silicone, a commonly used phantom material.^24^^,^^25^ The two-part silicone was mixed with nigrosin (Thermo Fisher Scientific, Waltham, Massachusetts), degassed and either injected into polytetrafluoroethylene (PTFE) tubing (Zeus Industrial Products Inc., Orangeburg, South Carolina) or poured into a Petri dish. After solidifying overnight, silicone phantoms were retrieved from molds. The small animal phantom was cut from silicone solidified in a Petri dish with $1.2\times 1.2\times 12\;{\mathrm{mm}}^{3}$ rectangular parallelepiped shape modeling the approximate diameter of a rat sciatic nerve and was designed to give a uniform light scattering and absorption profile so that the views could be easily merged. Next, we constructed the large animal nerve mimicking circular phantom that more closely approximated a large animal vagus nerve with internal structures like fascicles within a nerve. The 3.5-mm diameter 12-mm long PDMS cylinder was released from PTFE tubing. Six sutures were placed inside the phantom to mimic pig fascicle-sized structures.

In preparation for *in vivo* experiments, we also imaged *ex vivo* nerve samples (not presented in this paper). Finally, imaging small (rat) and large (minipig) animals using magnetic prism nerve holders demonstrated full *in vivo* operation and performance.

### 2.5 Subjects and Surgical Procedures

Both rat and minipig surgical procedures were approved by the Institutional Animal Care and Use Committee at the U.S. Food and Drug Administration, White Oak Campus.

The surgical procedure to expose the rat sciatic nerve was similar to one previously reported by Vasudevan et al.^1^ Briefly, the healthy Lewis rats (*Rattus norvegicus*, female, n=8, 8- to 16-weeks old, and 180 to 300 g) were placed under ketamine (75 mg/kg) dexmedetomidine (0.35 mg/kg) anesthesia, and the sciatic nerve was exposed and freed from surrounding tissue using microsurgical techniques. The prism nerve holder was gently slid under the nerve, and several PS-OCT imaging volumes were acquired. After the imaging session, animals were euthanized by administering intracardiac pentobarbital (200 mg/kg).

Healthy Yucatan minipigs (*Sus scrofa domesticus*, female, n=4, 6- to 8-month old, and 35 to 45 kg) were obtained from Sinclair Research (Auxvasse, Missouri). The minipig vagus nerves were exposed and stimulated following a procedure described by Nikolai et al.^26^ and Settell et al.^27^ The sedated animals were intubated on a surgical table and anesthetized using 1% to 4% isoflurane. Analgesia was achieved by intravenous (IV) infusion of fentanyl (2 to 100 mcg/kg/h). Anesthesia and analgesia were maintained by adjusting drug doses during the experiment to maintain a stable heart rate. On either side of the esophagus a 15- to 20-cm incision was made using an electrocautery blade and instrument (Aaron OR|Pro 300, Bovie Medical Corporation, Clearwater, Florida). After incision, blunt dissection was performed, carefully avoiding large branch vessels and cauterizing smaller ones, until the carotid sheath was reached. The vagus nerve was exposed and freed from the carotid sheath using blunt dissection and microsurgery techniques without damaging the carotid artery or internal jugular vein. After a 10- to 15-cm length of vagus nerve was exposed, the prism nerve holder was placed 1 cm caudal to the nodose ganglion by carefully sliding the prism nerve holder under the nerve [Fig. 3(c)] mimicking a typical placement of VNS electrodes in human subjects. To avoid mechanical stress, the surgeon used surgical bands while placing the nerve in the holder, similar to the technique used for cuff electrode implantation. Also the nerve was kept hydrated with a 0.9% sterile saline solution applied between image acquisitions. At the end of the experiment, the animal was euthanized using pentobarbital.

Sciatic nerves from rats or the vagus nerves from minipigs were excised after euthanasia, placed in 10% formalin over few weeks, sectioned, and preprocessed for histological staining. Paraffin-embedded sections were sliced, placed on glass slides, and triple stained against axon fibers (beta tubulin), myelin sheath (Anti-Myelin P0), and nuclei (4′,6-diamidino-2-phenylindole, DAPI). Immunohistochemistry (IHC) images were obtained on confocal microscope (FV3000, Olympus America Inc., Center Valley, Pennsylvania) using 10× (rat sciatic nerve) and 4× (minipig vagus nerve) objectives.

## 3 Results

### 3.1 Image Acquisition Using Tissue-Mimicking Phantoms

**Fig. 4:**
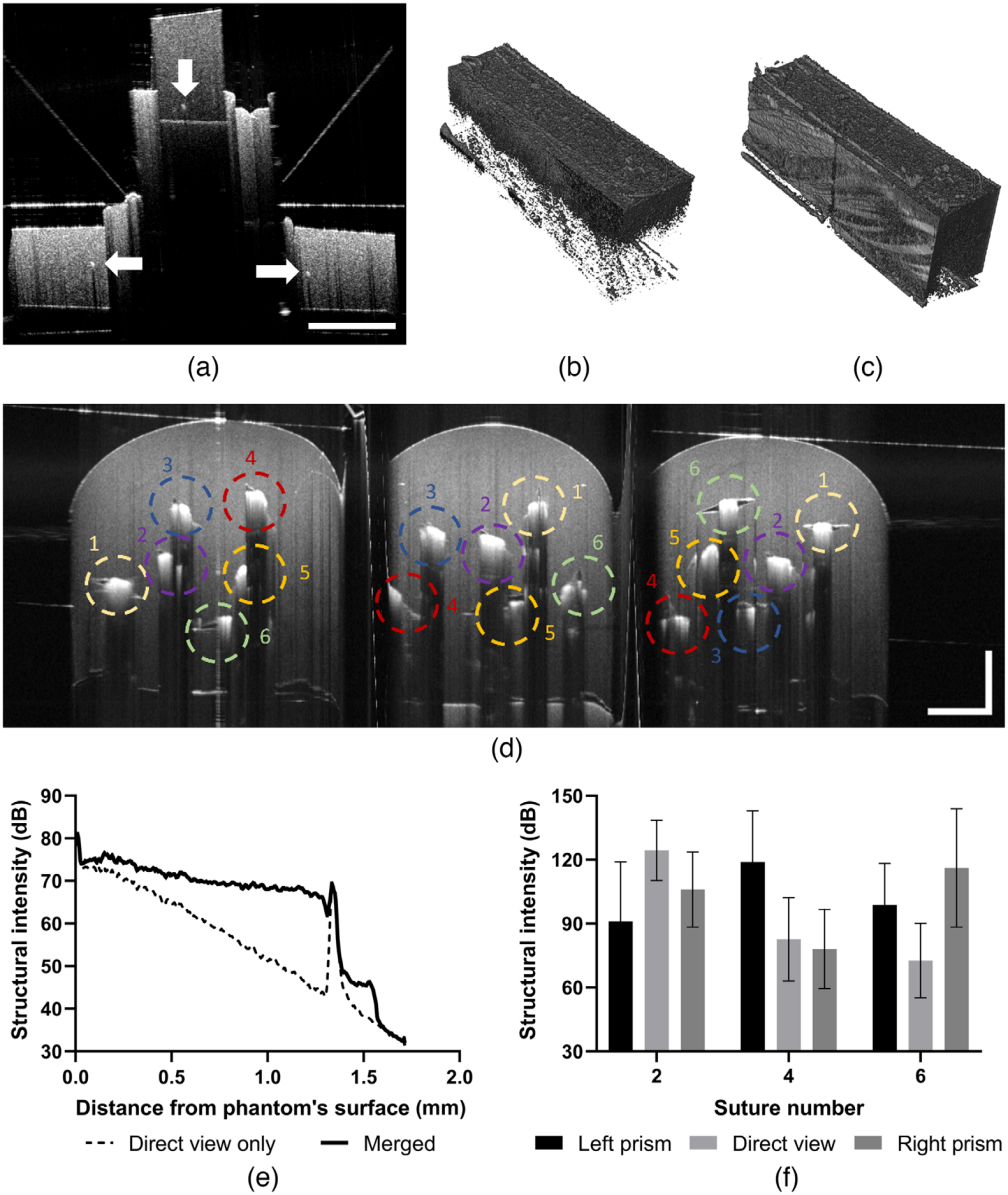
Structural OCT imaging of phantoms in prism nerve holder. (a) Similar features (white arrows) appear in the cross-sectional image of the small animal phantom both in (center) direct view and (left) projection images. Volumetric rendering of images (b) without and (c) with merged via maximization prism-reflected portion. Cross-sectional view of a large animal phantom in the prism nerve holder (d) from a structural volume that was acquired with the fast-scanning mirror aligned along the phantom, and the coherence gate position was set in the software to shift positions at the borders of the prisms. Numbered dashed colored circles represent the same suture in the prism and direct views. Averaged axial profile comparison (e) at the position of a bright feature (b, central arrow) demonstrates a 22-dB improvement in signal intensity at 1.25 mm depth for merged volume (c) compared to direct view volume (b). Images from prism views demonstrate >30-dB increase in intensity for sutures 4 and 6 (f) from the bottom half of the large animal phantom, while, as expected, the average intensity of suture 2 is larger in the direct view. Error bars represent standard deviation from suture’s region of interest. Scale bars: 1 mm.

Figure 4 shows images of the tissue-mimicking PDMS phantoms using the prism nerve holder [Figs. 4(a)–4(c): small animal nerve phantom and Fig. 4(d): large animal nerve phantom). The inclusion (hyperreflective spot) at the bottom of the small animal phantom can be seen in all three cross-sectional views—through both prisms and imaged directly from top. In order to merge the prism-reflected and direct back-reflected regions of the OCT scans, we developed custom software in MATLAB (Mathworks, Natick, Massachusetts). The current implementation is semiautomated and requires manual mapping of landmarks for merging. Averaged cross-sectional segments of the OCT scans are used for estimating projective geometrical transforms of prism regions. The final geometrical transform consists of 90-deg rotation, mirror reflection, and residual affine transform estimated by imregtform() function. These geometrical transforms are applied frame-by-frame to merge the prism-reflected and directly imaged volumes via maximization or alpha blending. Figure 4(e) demonstrates a 22-dB increase in signal intensity over 1.25-mm depth in the small animal phantom in the merged volume via maximization compared to the direct view volume.

Figure 4(d) demonstrates cross-sectional structural OCT images of a large animal phantom in a prism nerve holder with fast galvanometer scanning along the phantom and reference mirror change during volume acquisition. Light scattered from sutures produced a high-intensity OCT signal which in many instances shadowed the structures beneath the suture. The same sutures in direct view and prism views are marked with the same number and color. The side view prism images allowed visualization of not only sutures 4–6, which were partially shadowed on the direct view, but also finer details, such as tears, in PDMS due to suture placement. The average intensity of sutures 2 to 6 from the prism and direct view images are compared in Fig. 4(f) showing >30  dB increase for sutures 4 and 6 from the prism view images. The separation between the direct view and side prism view images was 6.5 mm with a fixed coherence gate position. By implementing the coherence gate change, all three subimages appear at the same axial position within the OCT image.

Tests with small (1.2 mm) and large (3.5 mm) tissue-mimicking phantoms demonstrated the increased light penetration without the need to mechanically adjust the imaging depth and verified that the geometrical parameters of the fabricated prism nerve holders are suitable for small and large animal peripheral nerve OCT imaging. Complementary information from the prism views provided fine structural details which were not accessible due to the limited penetration depth and highly scattering objects overshadowing structures below. However, functional OCT imaging modalities (OCTA and PS-OCT) require the presence of moving scatterers and birefringence,^28^ which adds more complications to phantom manufacturing and validation^29^ and is beyond the scope of this paper.

### 3.2 Small Animal (Rat) Imaging

**Fig. 5:**
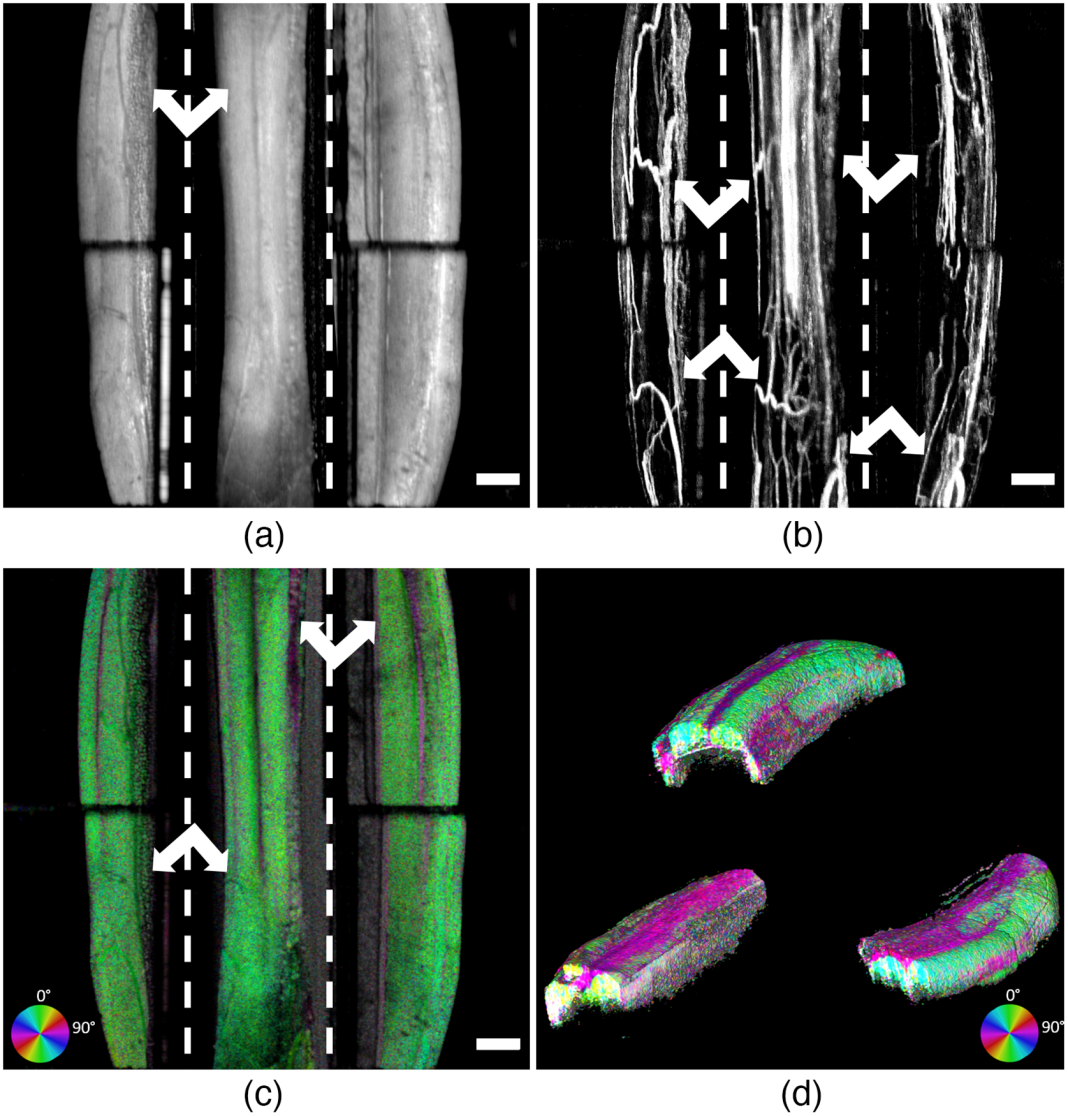
*En face* averaged OCT projections (a) structural OCT, (b) OCTA, (c) BwOA, and (d) BwOA volumetric reconstruction of *in vivo* PS-OCT rat sciatic nerve imaging using double-sided magnetic prism nerve holder. The dashed lines demonstrate the borders of prism regions. Imaging with prisms demonstrates features of adipose tissue (structural), blood vessels (OCTA), and large fascicles (BwOA), which were not accessible with top-bottom nerve imaging (white arrows). Plastic from the nerve holder channel under the nerve as well as reflection artifact from the mirror interface can be seen on the volumetric reconstruction as random noise coloring. Scale bars: 500  μm.

We used the double-sided magnetic prism nerve holder to demonstrate improved OCT imaging in rat sciatic nerve (Fig. 5). The images were obtained before full implementation of the coherence gate change, and as such a small OCT depth roll-off effect can be observed in the structural *en face* projection [Fig. 5(a)] of the prism sections, i.e., the structural image appears somewhat dimmer in the prism sections. Adipose tissue appears on the left prism projection and is not observable on the direct view. The fascicular margins manifest as dimmer structural OCT signals, but they are hard to distinguish from the vasculature which also has lower structural OCT signal. OCTA images collected with the prism nerve holder reveal portions of the vasculature that cannot be seen in the direct images alone [Fig. 5(b)]. Large blood vessel bifurcations can be easily observed and co-located between prism and direct views, while the capillary network from bottom half of the nerve can only be observed through the prism view [Fig. 5(b)]. BwOA imaging [Figs. 5(c) and 5(d)] provides clear discrimination between fascicles as the interfascicular epineurium PS-derived optical axis orientation (magenta, ∼40  deg) is almost orthogonal to fascicular optical axis orientation (green, ∼120  deg). The prism views provide information about a fascicle that was shadowed underneath the two fascicles visible on direct view. Multimodal (structural, OCTA, and BwOA) *en face* projections clearly demonstrate that images obtained from prism regions contain views of vascular and fascicular structure from the bottom half of the nerve that are not visible on direct view. Isometric projection of volume rendering of BwOA [Fig. 5(d)] provides an overview of acquired polarization-derived information from sciatic nerve in direct view and prism areas and also demonstrates the significant OCT depth difference between direct and prism view images without coherence gate adjustment.

**Fig. 6:**
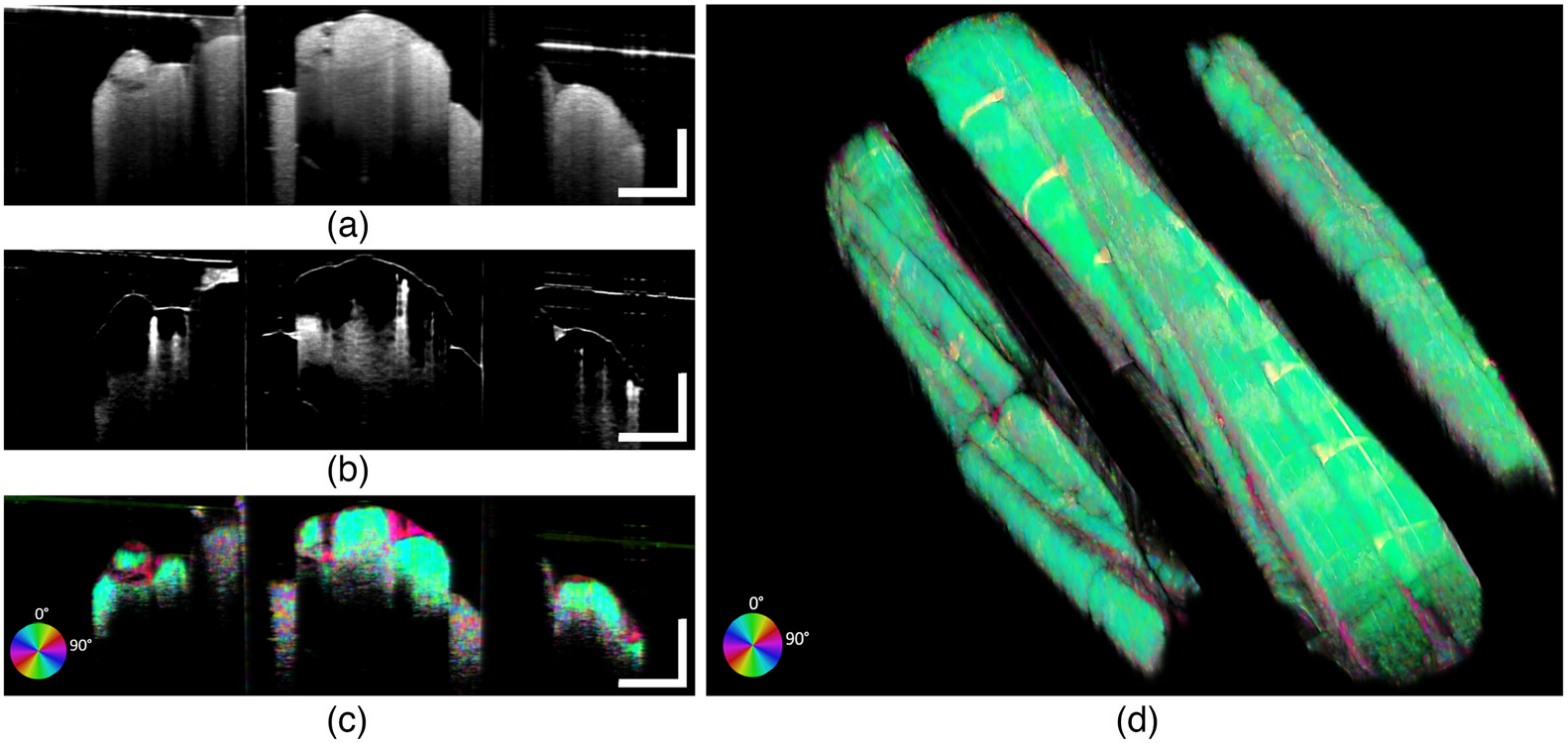
Cross-sectional (averaged five adjacent frames) OCT images (a) structural OCT, (b) OCTA, (c) BwOA, and (d) BwOA volumetric reconstruction of rat sciatic nerve. Scanning protocol was modified to perform fast scanning (x axis) along the nerve. In this configuration, B-scans are collected continuously during the slow scan (y axis) of the first set of prisms, nerve, and then second set of prisms with stepwise CG adjustment during volume acquisition. Scale bars: 500  μm.

Figure 6 shows OCT cross-sectional and volumetric images of the rat sciatic nerve with implementation of the CG shift to further minimize the effect of roll-off. The scanning axes are switched from the traditional orientation so that the fast scan axis is along the nerve and slow scan axis is across the nerve, and the reference mirror is shifted (in software) by 2 mm at two points between the prism and direct views.

### 3.3 Large Animal (Yucatan Minipig) Imaging

**Fig. 7:**
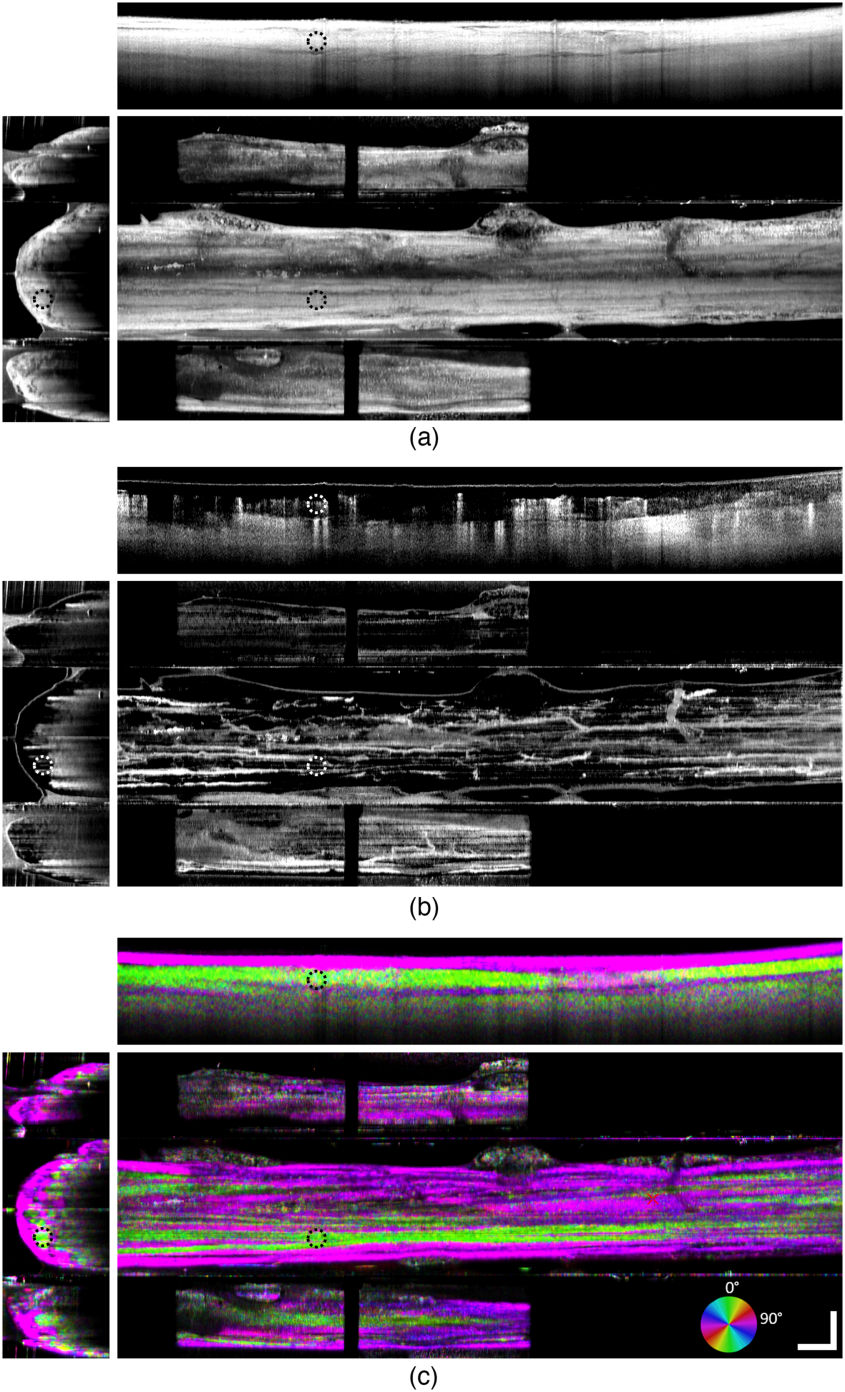
Multimodal OCT projections of a minipig vagus nerve imaged using the magnetic prism nerve holder. (a) Structural OCT, (b) OCTA, and (c) BwOA. Orthogonal projections were generated at the coordinates outlined by dashed circles located within a large fascicle. Scale bar: 1 mm.

Figure 7 presents orthogonal projections of structural OCT, OCTA, and BwOA volumes acquired *in vivo* from a minipig with the prism nerve holder using our PS-OCT system. Each image represents five averaged adjacent projections. Although fascicles can be seen on structural OCT [Fig. 7(a)] direct nerve images, structural OCT images from the mirror side views provide poor fascicular contrast and low image signal-to-noise ratio due to absorption and scattering in the presence of blood. However, such fascicles are clearly visualized in the BwOA images [Fig. 7(c)], where the difference in optical axis orientation between fascicles and collagenous epineurium is clearly observed. Additional information from the mirror side views of the nerve indicates fat and connective tissue (“bubbling” structures with no defined optic axis orientation) present on one side of the nerve. Our PS-OCT results [Fig. 7(c)] demonstrate that distinct fascicles in minipig vagus nerves provide lower retardation relative to epineural layers with high OCT signal intensity.

**Fig. 8:**
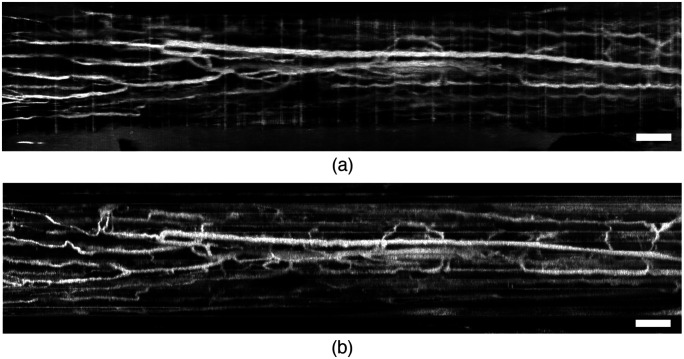
Comparison of *en face* OCTA projections of a minipig vagus nerve imaged *in vivo* using the magnetic prism nerve holder with orthogonal scanning strategies. (a) Fast scanning mirror scans the beam across the nerve and (b) B-scans acquired along the nerve. Scale bars: 1 mm.

*En face* OCTA projections [Fig. 7(b)] show a vast network of blood vessels running both along and across the nerve. Although some of the blood vessels visible through mirror prisms do not appear in the *en face* projection due to apparent difference in imaging depth, those blood vessels are visible in the cross-sectional images. Additional noise mitigation and motion suppression is achieved with a scanning protocol acquiring B-scans along the nerve. Figure 8 demonstrates the difference in OCTA imaging quality between mirror scanning protocols along the nerve and across the same nerve. Both volumes were acquired from identical FOVs.

## 4 Discussion

Deep tissue penetration and side visualization of peripheral nerves in living animals provides significant additional structural information—including nerve fascicle and blood vessel traces—to aid a variety of neurostimulation and neuromodulation applications. Although recent reports of *ex vivo* nerve fascicle tracing^30^ and fascicular structure^27^ have significantly advanced our understanding of the PNS, label-free imaging techniques are preferred for *in vivo* surgical applications. Currently, x-ray and computed tomography are routinely used to assess the implantation of stimulation electrodes postoperatively;^31^ however, these medical imaging modalities do not have high enough resolution to provide information on nerve fiber organization for targeted stimulation. High-frequency ultrasound has been demonstrated for intraoperative imaging of pig VN owing to its excellent penetration depth.^32^ However, ultrasound imaging lacks the required specificity for fascicle discrimination. On the other hand, multiphoton nonlinear optical microscopy has excellent resolution (<1  μm) and can discriminate between myelinated and unmyelinated axons, but is limited in penetration depth (∼100  μm) and FOV (∼a few hundred μm), and has other limitations related to medical device translation.^33^ Linear and nonlinear optical imaging techniques (e.g., three photon microscopy and optical coherence microscopy) in the 1.3-, 1.7-,^34^ and 2.2-μm^35^ water absorption windows have shown great progress in increasing the imaging penetration depth by navigating the balance between decreased scattering and increased water absorption with increased illumination wavelength. *In vivo* results in mouse brain demonstrated up to 1.2 mm penetration depth. However, the light scattering in peripheral nerves is higher than in brain, and there is limited availability of components for light delivery and detection at mid-infrared wavelengths. This currently creates a challenge for accessing PS-OCT derived biomarkers and translation of longer wavelength OCT systems for surgical applications.

Our PS-OCT system with 1300-nm central wavelength provides a compromise between penetration depth (∼700  μm), axial resolution (5.7  μm), lateral resolution (30  μm), field size (15  mm×20  mm), and imaging power (15 mW). Moreover, its multiple PS and OCTA channels are designed to collect myriad structural and functional details from tissue targets. The adjunctive use of a magnetic prism holder provides side imaging views of large animal peripheral nerves with deep penetration of several millimeters revealing additional anatomical and functional features.

Optimal visualization of tissue targets using the prism nerve holder would be enhanced by processing software to automatically merge the top-down (direct) and side (prism) views, initially subsequent to acquisition but eventually in real time with the aid of parallel processing hardware (e.g., GPUs) or machine learning algorithms. We made initial progress toward this goal with a software algorithm that uses a geometric transformation between landmarks manually chosen in one B-scan between top-down and side views, which is then applied with alpha blending to all B-scan in a set of multimodal (structural, OCTA, and BwOA) volumes. The merging algorithm works reasonably well on small animal nerves; however, large animal nerves present larger distortions due to refractive index differences between air and tissue.^36^ The larger the merging volume is, the larger the absolute error of pixel positioning transforms from prism to direct view is. Additionally, a significant amount of connective and adipose tissue present in large animal nerves adds inhomogeneity in the refractive index compared to small animal nerves. A typical geometric transform solver cannot resolve such distortion, and special consideration is needed to fully automate merging direct and prism subvolumes for large animal nerves. Tail artifacts pose another complication for applying a merging algorithm to OCTA volumes. After merging, the same blood vessel appears with three tail artifacts. Methods have been previously demonstrated by other groups to remove OCTA tail artifacts,^37^^,^^38^ which could be applied prior to merging OCTA volumes. All of these issues do not appear when *ex vivo* tissue images are merged because excised tissue would normally be imaged either through a glass slide or in a bath with refractive index matching fluid.^39^ Index matching is not feasible during surgery because any additional liquid in the surgical pocket is contaminated with red blood cells which in turn significantly degrade the OCT scan quality due to excessive scattering and water absorption. Despite our best effort to remove excessive liquid pooling in the nerve holder channel after the rehydration, some of our acquired volumes suffered signal degradation due to liquid trapped in the meniscus between the nerve and the prisms. In this paper, our *in vivo* peripheral nerve results are displayed in separate volumes and refining and validating a merging algorithm is left for a future study.

Anecdotally, our method of encoding tissue optical axis orientation with birefringence into multicolor images shows that some of the fascicles exhibit different optic axis orientation values. This can be observed in the bottom panels of Fig. 7, where cross-sectional BwOA projections of some fascicles appear green, whereas others appear magenta, corresponding to a ∼90-deg difference of optic axis orientation. We observed similar findings in other minipig experiments (n=4), however, a separate study is required to establish any putative correlation between PS-OCT signal and fascicular composition.

The deep tissue imaging strategy presented here provides complementary information on PS-OCT derived imaging modalities using side views of a tissue target. The current prism nerve holder implementation does not disrupt the standard PS-OCT imaging path, does not require extensive hardware modifications,^40^ custom optics,^41^ or advanced image processing,^42^ and relies only on off-the-shelf components and those fabricated by 3D printing. These advantages allowed us to transfer this technology to our research collaborators for their experimental use. Furthermore, our hardware approach could eventually be used in addition to beam shaping strategies and advanced image processing techniques to further improve the quality of 3D structural and functional peripheral nerve assessment.

## 5 Conclusion

We developed a prism-based peripheral nerve holder, imaging protocol, and postprocessing merged visualization software to provide enhanced penetration for PS-OCT imaging. We demonstrated the successful application of this approach on tissue-mimicking phantoms, *in vivo* rat sciatic nerves, and *in vivo* minipig vagus nerves. The prism nerve holder will improve OCT imaging of peripheral nerves and enhance the development of imaging-based biomarkers to prevent nerve injury during the treatment of various neurological conditions and other neuromodulation applications.

## Code, Data, and Materials Availability

The data underlying the results presented in this paper are available from the authors upon reasonable request.
